# Histomorphometric analysis of liver biopsies of treated patients with Gaucher disease type 1

**DOI:** 10.4322/acr.2021.306

**Published:** 2021-08-20

**Authors:** Rodrigo Tzovenos Starosta, Marina Siebert, Filippo Pinto e Vairo, Bruno Lafaiete de Lima Costa, Christiano Tomaso Ponzoni, Ida Vanessa Doederlein Schwartz, Carlos Thadeu Schmidt Cerski

**Affiliations:** 1 Universidade Federal do Rio Grande do Sul, Graduate Program in Genetics and Molecular Biology, Porto Alegre, RS, Brasil; 2 Washington University, Department of Pediatrics, Saint Louis, MO, USA; 3 Hospital de Clínicas de Porto Alegre, Laboratorial Research Unit, Experimental Research Center, Porto Alegre, RS, Brasil; 4 Universidade Federal do Rio Grande do Sul, Graduate Program in Science in Gastroenterology and Hepatology, Porto Alegre, RS, Brasil; 5 Mayo Clinic, Center for Individualized Medicine, Rochester, MN, USA; 6 Mayo Clinic, Department of Clinical Genomics, Rochester, MN, USA; 7 Hospital de Clínicas de Porto Alegre, Department of Surgical Pathology, Porto Alegre, RS, Brasil; 8 Universidade Federal do Rio Grande do Sul, Department of Genetics, Porto Alegre, RS, Brasil; 9 Hospital de Clínicas de Porto Alegre, Medical Genetics Service, Porto Alegre, RS, Brasil

**Keywords:** Gaucher Disease, Image Cytometry, Hepatocytes, Bile Canaliculi, Biopsy, Large-Core Needle

## Abstract

Gaucher disease (GD) is an autosomal recessive lysosomal disorder caused by a disturbance in the metabolism of glucocerebroside in the macrophages. Most of its manifestations – hepatosplenomegaly, anemia, thrombocytopenia, and bone pain – are amenable to a macrophage-target therapy such as enzyme replacement. However, there is increasing evidence that abnormalities of the liver persist despite the specific GD treatment. In this work, we adapted histomorphometry techniques to the study of hepatocytes in GD using liver tissue of treated patients, developing the first morphometrical method for canalicular quantification in immunohistochemistry-stained liver biopsies, and exploring histomorphometric characteristics of GD. This is the first histomorphometric technique developed for canalicular analysis on histological liver biopsy samples.

## INTRODUCTION

Gaucher disease (GD) is one of the most common lysosomal disorders with an estimated prevalence of 1:60,000 in the general population and 1:800 in the Ashkenazi Jewish population.^1^ The pathophysiology of GD is classically defined as a disturbance in the processing of sphingolipids inside the macrophages,^1^^,^ 2 the main manifestations of GD being a function of dysregulated macrophagic activation^2^^,^ 3 and invasion of tissues by Gaucher cells.^4^^,^ 5 Macrophage-targeted therapy through infusions of recombinant glucocerebrosidase (GCase) which is uptaken via the mannose receptor pathway^6^ has been successful in alleviating the key clinical findings in GD – anemia, thrombocytopenia, hepatosplenomegaly – and improving quality of life in these patients.^7^ However, there are still features of GD that are not fully explained by the involvement of macrophages. It is known that patients with GD, even after long-term treatment, tend to have a higher liver stiffness (which is a surrogate measurement for fibrosis) than controls.^8^^-^ 11 Moreover, the biliary phenotype of GD has been subject of increasing focus: patients with GD have an increased incidence of gallstones,^12^^-^ 15 and an abnormal bile composition with increased level of glucocerebroside (glucosylceramide; GlcCer) and other sphingolipids such as glucosylsphingosine (GlcSph, alias lyso-GL1).^12^^,^ 16 Biliary excretion of GlcCer is being suggested as a protective factor against hepatocellular storage of this substance;^16^^,^ 17 however, this export of lysosomal GlcCer into the bile canaliculi may be one of the possible mechanisms of injury to the biliary system in GD through interference with the composition of bile and the function of transporters in the hepatocytic apical membrane. This interaction between GlcCer and bile transporters has been demonstrated in studies of the same transporters in cancer multidrug resistance.^18^^,^ 19

Histomorphometry, or histological morphometry, is the quantification of morphology at the tissue level. It is a well-established technique with applications in the research of many tissues, including the liver, where it has been used for the quantification of characteristics such as fibrosis, immunohistochemical markers, and steatosis.^20^^,^ 21 ^,^ 22 It has also been used as a grading and prognostic marker in a variety of tumors.^23^^-^ 25

Histomorphometry is still an underutilized, incipient technique in canalicular pathology^26^. Although some studies have used morphometric parameters such as canalicular length and total canalicular area in a research context, making use of tools such as immunofluorescence and transmission electron microscopy 27 ^,^ 28, there are no studies on the use of histomorphometry in clinical liver samples. In this study, we adapted histomorphometry to the study the canalicular parameters in liver biopsies of patients with GD.

## METHODOLOGY

### Samples

We analyzed liver biopsies of patients with GD followed at the Gaucher Reference Center of the Hospital de Clínicas de Porto Alegre (GRC-HCPA). The samples were preserved in paraffin and were retrieved from the archive of the Service of Surgical Pathology (SSP) of HCPA. The archive of the SSP was also searched for liver biopsy samples with a diagnosis of “healthy liver tissue” or “steatohepatitis grade 1” ("clinical liver disease") by an expert in Liver Pathology (CTSC).

Samples were processed and stained with immunohistochemistry (IHC) for CD10 (to highlight the bile canaliculi) according to the SSP protocol. Briefly, samples were cut into 3 µm-thick sections and deparaffinized. Antigen recovery was performed with CC1 buffer at pH 9.0 and 95ºC for 2 minutes followed by peroxidase blocking with OptiView Peroxidase Inhibitor (Ventana Medical Systems). The primary rabbit anti-human anti-CD10 monoclonal antibody (clone SP67; Roche Diagnostics, Tucson, Arizona, USA) was incubated for 28 minutes at 36ºC. After primary antibody incubation, the reaction was detected with the OptiView DAB IHC Detection Kit (Ventana Medical Systems) and slides were counterstained with hematoxylin and bluing reagent (Li_2_CO_3_ + Na_2_CO_3_).

### Imaging

Stained and mounted slides were microphotographed with the CellSens software (Olympus Corporation) at a magnification of 1000x. Each slide had 6 random high-power fields captured and saved as tagged image file format (.tiff) images.

### Histomorphometry

**Figure 1:**
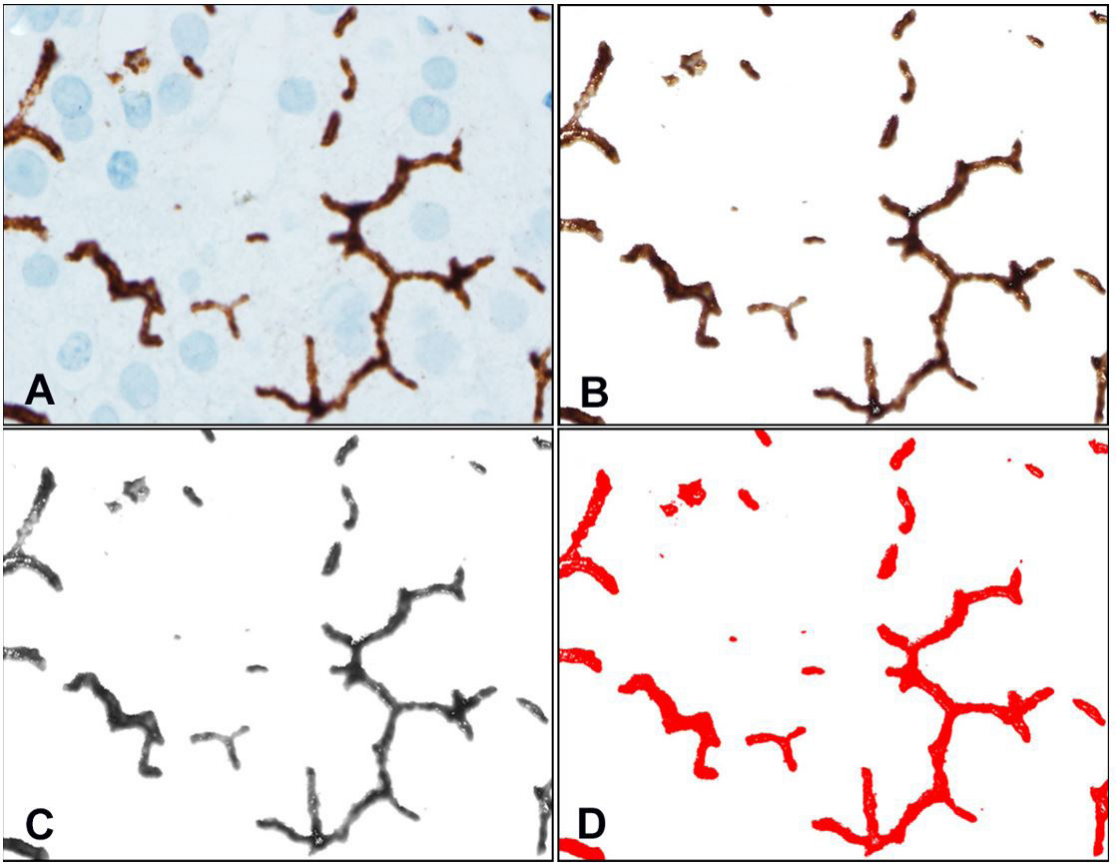
A – RGB photomicrograph, 1000x, showing IHC staining anti-CD10 highlighting the canaliculi in brown; B – extraction of canaliculi from background using the IHC Toolbox plugin on ImageJ; C – Transformation of extracted canaliculi from RGB to 8-bit; D – Thresholding of 8-bit image selecting all IHC-positive areas, with no overlap with the background.

Each stained image was converted from the native RGB color format to 8-bit using the ImageJ software.^29^ The pixel-to-µm conversion was calculated from the scale generated by CellSens. For canalicular histomorphometry, IHC-stained RGB images were treated with the IHC Toolbox plugin (https://imagej.nih.gov/ij/plugins/ihc-toolbox) to isolate IHC-positive areas (i.e., hepatic canaliculi), as shown in Figure 1.

**Figure 2:**
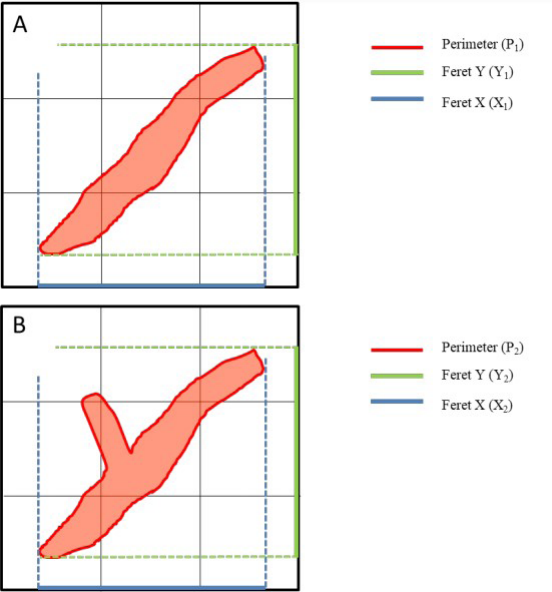
A – schematic depiction of a less-branching canaliculus, for clarification of the perimeter-to-Feret ratio. The solid red line is the canaliculus perimeter (P_1_). The solid green line represents the projection of the canaliculus on the Y axis of the image field, which is the Feret Y (Y_1_) measure. The solid blue line represents the projection of the canaliculus on the X axis of the image field, which is the Feret X measure (X_1_). The Feret diameter of a canaliculus (F_1_) is the arithmetic mean of X_1_ and Y_1_; B – schematic depiction of a more-branching canaliculus. The solid red line is the canaliculus perimeter (P_2_). The projection of this canaliculus in the Y (Y_2_) and X (X_2_) axes are depicted as the solid green and blue lines, respectively, and the Feret diameter of this canaliculus (F_2_) is the arithmetic mean of X_2_ and Y_2_. As shown, the increase in branching affects more the perimeter than the Feret diameter of a canaliculus: P_1_ < P_2,_ F_1_ ≈ F_2_. In this way, P_1_/F_1_ < P_2_/F_2_. These images are theoretical simplified schemes for clarification and are *per se* not representative of either group.

Manual thresholding was used to select all IHC-positive regions of interest (ROIs). ROIs with <10 pixels of area were considered to be artefactual and were excluded from the histomorphometric analysis. Each ROI was then analyzed for area in µm^2^, mean gray value (MGV), perimeter in µm, Feret diameter in µm, and solidity. Feret diameter is defined as the mean measure of the projection of an object to orthogonal tangential axes. The perimeter-to-Feret ratio was calculated from obtained values and was used as a measure of canalicular branching, as detailed in Figure 2.

MGV was corrected to absorbance (corrected MGV, "cMGV") with the formula cMGV = 255 – MGV. Due to the background correction obtained with the IHC Toolbox plugin, canalicular cMGV was not normalized. Because of the pattern similarity between the hepatic canaliculi and the bone trabeculae, we used the Map_BoneMicrostructure plugin (https://imagej.nih.gov/ij/plugins/microstructure) to obtain mean canaliculi thickness in µm for whole images.

### Statistical Analysis

Because of the small sample sizes, all variables were considered parametric for statistical analysis. For descriptive statistics, variables are described as mean ± SD. The Student's T-test was used for comparisons between group means. A subgroup analysis was conducted *post-hoc* to the group comparisons. For this analysis, subjects were divided into those with clinical liver disease (steatohepatitis or cirrhosis) and those without. Analytic statistics were not performed in the subgroup analysis due to the small sample sizes.

### Ethical Approval

This study was approved by the HCPA Research Ethics Committee under the number #18-0654. Research consent was waived by the HCPA Research Ethics Committee because of the retrospective nature of the analyses.

## RESULTS

### Patients

Liver biopsy samples of five type 1 GD patients were retrieved. Patient characteristics are displayed in Table 1. Two patients (pts 3 and 4) had clinical liver disease. Liver biopsy samples of seven controls were retrieved (healthy = 4; steatohepatitis = 3).

**Table 1:** Characteristics of GD patients

Patient	Sex	*GBA* Genotype	Age at biopsy (y)	Treatment at biopsy (months)	Biopsy diagnosis
1	F	p.Glu388Lys/p.Ser405Asn	48	Miglustat (11)	Macrovesicular steatosis
2	F	p.Asn409Ser/p.Leu483Arg	63	Miglustat (22)	Hemosiderosis, presence of Gaucher cells
3	M	p.Asn409Ser/Rec*Nci*I	56	Imiglucerase 30 IU/Kg/biweekly (69)	Steatohepatitis with mild activity
4	M	p.Asn409Ser/Rec*Nci*I	61	Imiglucerase 30 IU/Kg/biweekly (72)	Cirrhosis, hemosiderosis, presence of Gaucher cells
5	M	p.Asn409Ser/Rec*Nci*I	42	Taliglucerase alfa 30 IU/Kg/biweekly (216)	Macrovesicular steatosis

All patients are diagnosed with GD type 1. Y = years-old; F = female; M = male; Rec*Nci*I = recombinant allele with the *GBA1* pseudogene, includes the p.Leu483Arg, p.Ala495Pro, and p.Val499Val variants.

### Canalicular Histomorphometry

Results of histomorphometrical analysis are displayed in Table 2. No significant statistical difference was found between the groups for any of the variables. The closest parameter to statistical significance was the Perimeter-to-Feret ratio (*p*=0.06).

**Table 2:** Results of histomorphometrical analysis.

Parameter	Gaucher disease			Control group			
	Total (n=5)	Clinical liver disease (n=2)	No clinical liver disease (n=3)	Total (n=7)	Clinical liver disease (n=3)	Healthy (n=4)	*p*-value
Area (µm^2^)	8.21 ± 4.76	7.71 ± 3.33	8.52 ± 6.27	8.23 ± 6.30	2.96 ± 1.05	12.19 ± 5.48	0.639
cMGV	114.29 ± 31.67	97.96 ± 4.59	125.18 ± 39.39	113.62 ± 47.67	65.48 ± 11.30	149.72 ± 20.14	0.876
Perimeter (µm)	11.42 ± 2.17	12.33 ± 3.81	10.81 ± 0.92	12.77 ± 6.36	7.98 ± 1.93	16.36 ± 6.19	0.870
Feret diameter (µm)	3.52 ± 0.73	3.83 ± 1.09	3.31 ± 0.56	3.46 ± 1.95	1.86 ± 0.47	4.65 ± 1.73	0.085
Perimeter-to-Feret ratio	2.89 ± 0.20	2.83 ± 0.03	2.93 ± 0.27	2.94 ± 0.05	2.95 ± 0.05	2.93 ± 0.05	0.060
Solidity	0.75 ± 0.03	0.76 ± 0.01	0.74 ± 0.04	0.73 ± 0.04	0.68 ± 0.005	0.76 ± 0.007	0.343
Thickness (µm)	8.69 ± 1.34	9.24 ± 1.31	8.32 ± 1.50	8.46 ± 1.48	9.49 ± 0.94	7.10 ± 0.60	0.793

The p-values are a result of the comparison between Gaucher disease (total) and control group (total). No analyses were performed for the subgroups.

## DISCUSSION

Histomorphometry is a digital image analysis approach that relies on the identification and analysis of morphological elements in a histological section.^30^ It is a technique used for diagnosis of neoplasms and to aid in tailoring cancer treatment.^31^ We used histomorphometry to explore canalicular parameters in a sample of patients with GD type 1.

Although no statistically significant differences were found in this study, the borderline *p*-value for the Perimeter-to-Feret ratio is promising, and, if confirmed in further studies with bigger populations, might indicate canalicular dysfunction as part of the pathogenesis of GD.

On subgroup analysis, patients with GD and no clinical liver disease seemed to have a reduced canalicular area, cMGV, perimeter, and Feret diameter than healthy controls – however, because of statistical analyses not being possible due to the small sample size, conclusions from the subgroup analysis are limited.

In contrast with the common cholestatic pattern of many diseases which consists of canalicular dilation,^32^ reduced branching has not been studied as much due to the lack of methods to perform a detailed analysis of these structures. It is known that patients with GD have increased secretion of GlcCer in bile, leading to physiological changes such as upregulation of *GBA2*, coding for a bile acid 3-O-glucosidase that can also metabolize GlcCer and GlcSph,^33^ producing toxic compounds such as sphingosine.^34^ It is possible that this process leads to biliary injury, thus impacting on the normal canalicular structure.

The main limitation of this study is the small sample size. GD is a rare metabolic disorder, and with the current technology available in clinical practice for follow-up of these patients – such as transient elastography and magnetic resonance imaging – liver biopsies are seldom performed because of the invasiveness of the procedure and the increased bleeding risk in this group.

In summary, this is the first report of the application of histomorphometry in the study of liver canaliculi in a metabolic disorder. A new parameter for canalicular analysis, the perimeter-to-Feret ratio, was developed and demonstrated. Although significant differences were not found, this study paves the way for further investigation of canalicular pathology in GD and in other diseases.

## CONCLUSION

No significant differences were found between GD and control samples. An almost-significant *p-*value was found for perimeter-to-Feret ratio, indicating that further exploring this new parameter in larger samples might yield valuable results. This is the first histomorphometric technique developed for canalicular analysis on routine liver biopsy samples.

### Research Highlights

• The perimeter-to-Feret ratio is informative on canalicular branching on liver biopsy samples.

• Canalicular parameters are amenable to quantification by histomorphometry on liver biopsy samples.
